# Effects of exercise on cervical muscle strength and cross-sectional area in patients with thoracic hyperkyphosis and chronic cervical pain

**DOI:** 10.1038/s41598-021-83344-4

**Published:** 2021-02-15

**Authors:** Hyunghun Moon, Sung-Ki Lee, Won-Moon Kim, Yong-Gon Seo

**Affiliations:** 1 Department of Sports Medicine, Cha University, 120, Haeryong-ro, Pocheon-si, Gyeonggi-do 11160 Republic of Korea; 2 Department of Sports Science, Dongguk University, 123, Dongdae-ro, Gyeongju-si, Gyeongsangbuk-do Republic of Korea; 3 Division of Sports Medicine, Department of Orthopedic Surgery, Samsung Medical Center, Sungkyunkwan University School of Medicine, 81 Irwon-ro, Gangnam-gu, Seoul, 135-710 Republic of Korea

**Keywords:** Diseases, Medical research

## Abstract

There is a lack of studies comparing the effects of different exercise types in patients with thoracic hyperkyphosis. Twenty-four subjects were divided into three groups: corrective exercise, resistance exercise, and physical therapy. The groups performed their respective interventions, two times per week for three months. Clinical outcomes, including the value of Cobb’s angle, cervical muscle strength and endurance, and the cross-sectional area of the cervical deep muscles were measured pre- and post-intervention. There was a significant difference in the changes in the thoracic Cobb’s angle between the groups (*P* < 0.001). The corrective exercise group revealed a significantly superior increase in muscle strength and endurance between pre- and post-intervention (*P* < 0.012). There was a significant difference in the cross-sectional area of the cervical deep muscles included longus capitis and multifidus between the groups (*P* < 0.036 and 0.007, respectively). The corrective exercise group showed the most significant increase in cross-sectional area between pre- and post-intervention (*P* < 0.012). A corrective exercise program is a more effective intervention than traditional resistance exercise and physical therapy for improving the thoracic Cobb’s angle, cervical muscle strength and endurance, and the cross-sectional area of the deep muscles in patients with thoracic hyperkyphosis.

Trial registration: KCT0005292.

## Introduction

The spinal curve facilitates body movement through the interaction of joints including the cervical, thoracic, lumbar, sacral, and hip joints, and enables maintaining upright posture while walking against the center of gravity of the human body^1^. The normal spinal curvature in the sagittal plane comprises a lordotic curve formed in the cervical and lumbar regions, and a kyphotic curve in the thoracic and sacral regions. Excessive curvature is defined as an angle of kyphosis ≥ 40° of the thoracic spine in the sagittal plane, commonly referred to as thoracic hyperkyphosis^2^.

Thoracic hyperkyphosis can cause a compensatory pattern in body movements, contributing to malalignment of the normal spinal curvature, and resulting in musculoskeletal problems such as back pain and dysfunction^3^. An increase in the kyphotic curve is related to malalignment of the cervical curve, such as hyperextension of the upper cervical region and hyperflexion of the lower cervical region, and continuous overload on the cervical vertebrae can cause forward head posture^4^. Forward head posture is associated with the shortening of the cervical extensors and weakness of the cervical flexors, leading to cervical pain due to continuous tension in the peri-cervical region^1^.

Exercise therapy is commonly recommended as an intervention to improve hyperkyphotic curve and physical function in thoracic hyperkyphosis^5,6^. Bautmans et al.^7^ reported that manual therapy for rehabilitation is an effective intervention to improve thoracic function in patients with thoracic kyphosis. Exercise types in previous studies include back extensor strengthening, abdominal strengthening, and postural education, among which the back extensor strengthening exercise is the most common intervention^5^. Progressive back strengthening exercise contributes to a significant decrease in thoracic kyphosis and increase in muscle strength and endurance^8^. Katzman et al.^6^ reported that thera-band exercise for enhancing strengthening is beneficial intervention to reduce angle of thoracic hyperkyphosis.

Various exercise intervention have been reported for thoracic hyperkyphosis, and corrective exercise also was reported as an intervention method attributed to improve spinal posture, balance, and well-being in older women with thoracic hyperkyphosis^9^. Corrective exercise included resistance training, stretching, and posture education is associated with reduction of kyphosis angle and thoracic posture^10,11^. According to previous studies, the different types of exercise prescriptions are associated with different clinical outcomes for improving hyperkyphotic curve, muscle strength, and quality of life^10,12,13^. However, most of studies reported have focused on reduction of thoracic kyphosis and improvement of muscle strength and the studies of comparison of effectiveness between different exercise interventions have not reported.

Therefore, this study aimed to investigate the effects of different exercise types on the thoracic Cobb’s angle, and cervical muscle strength and endurance, and to confirm an increase in the cross-sectional area (CSA) of the cervical muscles. Finally, this study aimed to determine the most optimal exercise type for enhancing cervical muscle strength and endurance in patients with thoracic hyperkyphosis with chronic cervical pain.

## Results

The characteristics of the subjects in this study did not show significant differences in baseline data (Table 1).

**Table 1:** Characteristics of study subjects.

Characters	Two active interventions		One passive	*P*
	CEG	REG	CG	
Numbers	8	8	8	–
Sex (female/male)	8/0	8/0	8/0	–
Age (years)	53.38 ± 5.61	50.00 ± 4.00	54.25 ± 6.16	.259
Height (cm)	158.85 ± 4.29	159.64 ± 4.19	159.16 ± 5.32	.996
Weight (kg)	57.40 ± 5.56	57.41 ± 3.66	56.68 ± 4.44	.941
Body mass index (kg/m^2^)	22.73 ± 1.69	22.59 ± 2.06	22.44 ± 2.34	.910
Neck pain (score)	7.50 ± 0.54	7.63 ± 1.06	7.25 ± 0.46	.588

Data are means ± standard deviation. CEG, corrective exercise group; REG, resistance exercise group; CG, control group.

*P* values are from Kruskal–Wallis test.

In result of Cobb's angles, there was a significant difference in the changes in the Cobb’s angle of the thoracic curve between the groups (Kruskal–Wallis test, *P* < 0.000). According to the results of post hoc test, CEG showed a significantly higher than REC and CG (Mann–Whitney U test; CEG vs. REG, *P* < 0.007; CEG vs. CG, *P* < 0.001). REG was showed more improvement than CG (*P* < 0.003). The two groups with active interventions, CEG and REG, showed a significant improvement (Wilcoxon singed rank test, *P* < 0.012, *P* < 0.011, respectively), while CG did not show a significant difference between these periods (*P* < 0.317).

In result of the maximal cervical muscle strength, there was a significant difference in the changes between the groups (Kruskal–Wallis test, *P* < 0.038). The two active intervention groups, CEG and REG, had a significant increase between pre- and post- intervention (Wilcoxon signed-rank test, *P* < 0.012 and *P* < 0.012, respectively) but not revealed a significant difference in CG (*P* < 0.231). The results of Mann–Whitney U test for post hoc test, there was no significant difference between the group (CEG vs. REG, *P* < 0.14; CEG vs. CG, *P* < 0.031; REG vs. CG, *P* < 0.073).

**Figure 1:**
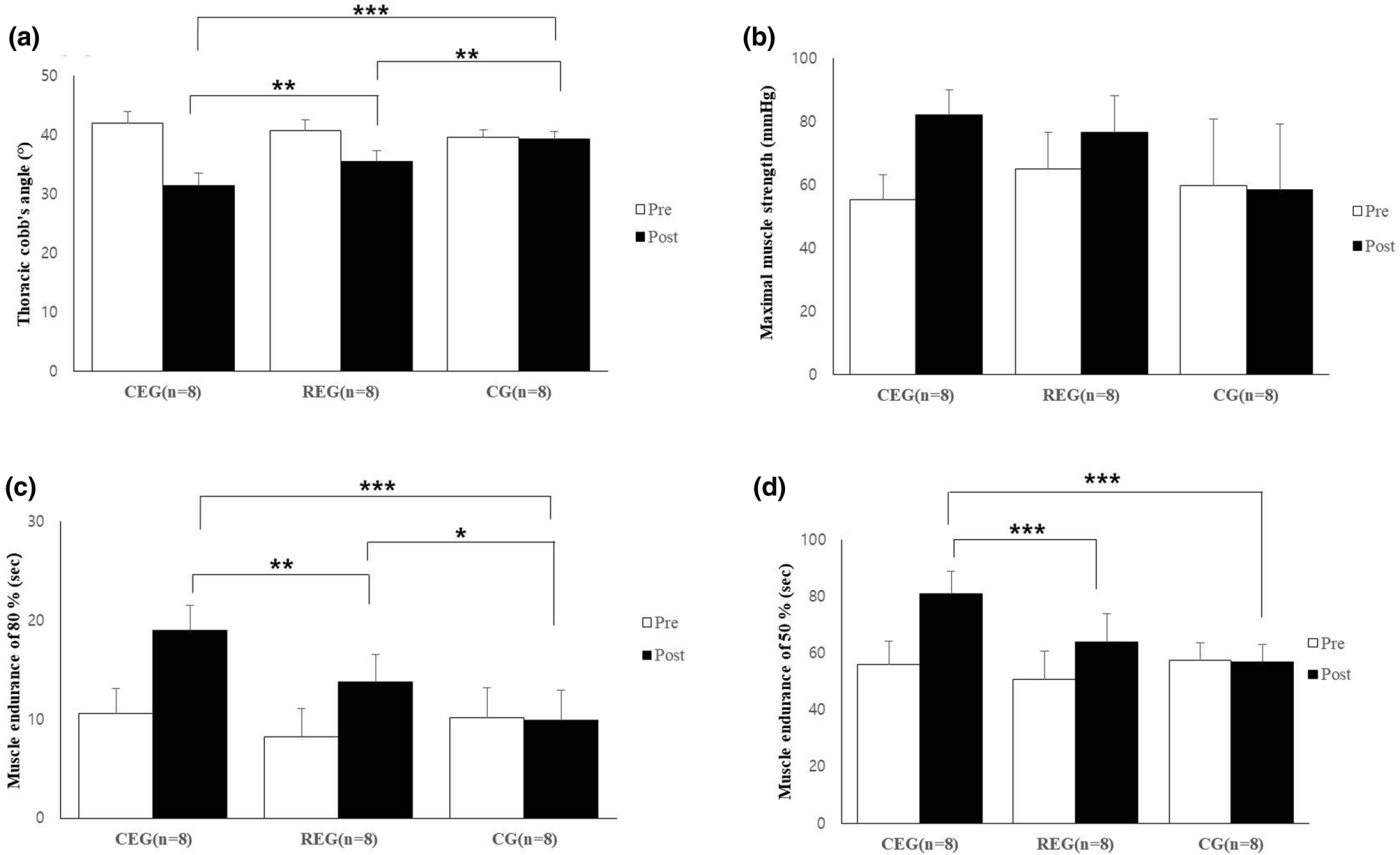
The difference of thoracic angle and cervical muscles strength and endurance after intervention. (**a**) Thoracic Cobb’s angle; (**b**) Maximal muscle strength; (**c**) Muscle endurance of 80%; (**d)** Muscle endurance of 50%. CEG, corrective exercise group; REG, resistance exercise group; CG, control group. *P* < 0.0167*, *P* < 0.00167**: the results from Bonferroni correction.

In the muscle endurance of 80% and 50%, the results revealed that there was a significant difference in the change between the groups (Kruskal–Wallis test, *P* < 0.000). In the results of post hoc test, CEG showed a higher improvement than REG and CG (Mann–Whitney U test, *P* < 0.004 and *P* < 0.001, respectively) and REG was more improved than CG (*P* < 0.015) in muscle endurance of 80%. In result of Wilcoxon singed-rank test, CEG and REG has showed a significant improvement after intervention (*P* < 0.011 and *P* < 0.012, respectively) but not improved in CG (*P* < 0.914) (The change of 50% muscle endurance in CEG showed also higher than REG and GC (Mann–Whitney U test, *P* < 0.001 and *P* < 0.001, respectively) but not showed a significant difference between REG and CG (*P* < 0.035). In results of Wilcoxon signed-rank test, CEG and REG has showed a significant improvement after intervention (*P* < 0.011 and *P* < 0.021, respectively) but not improved in CG (*P* < 0.667) (Fig. 1).

**Figure 2:**
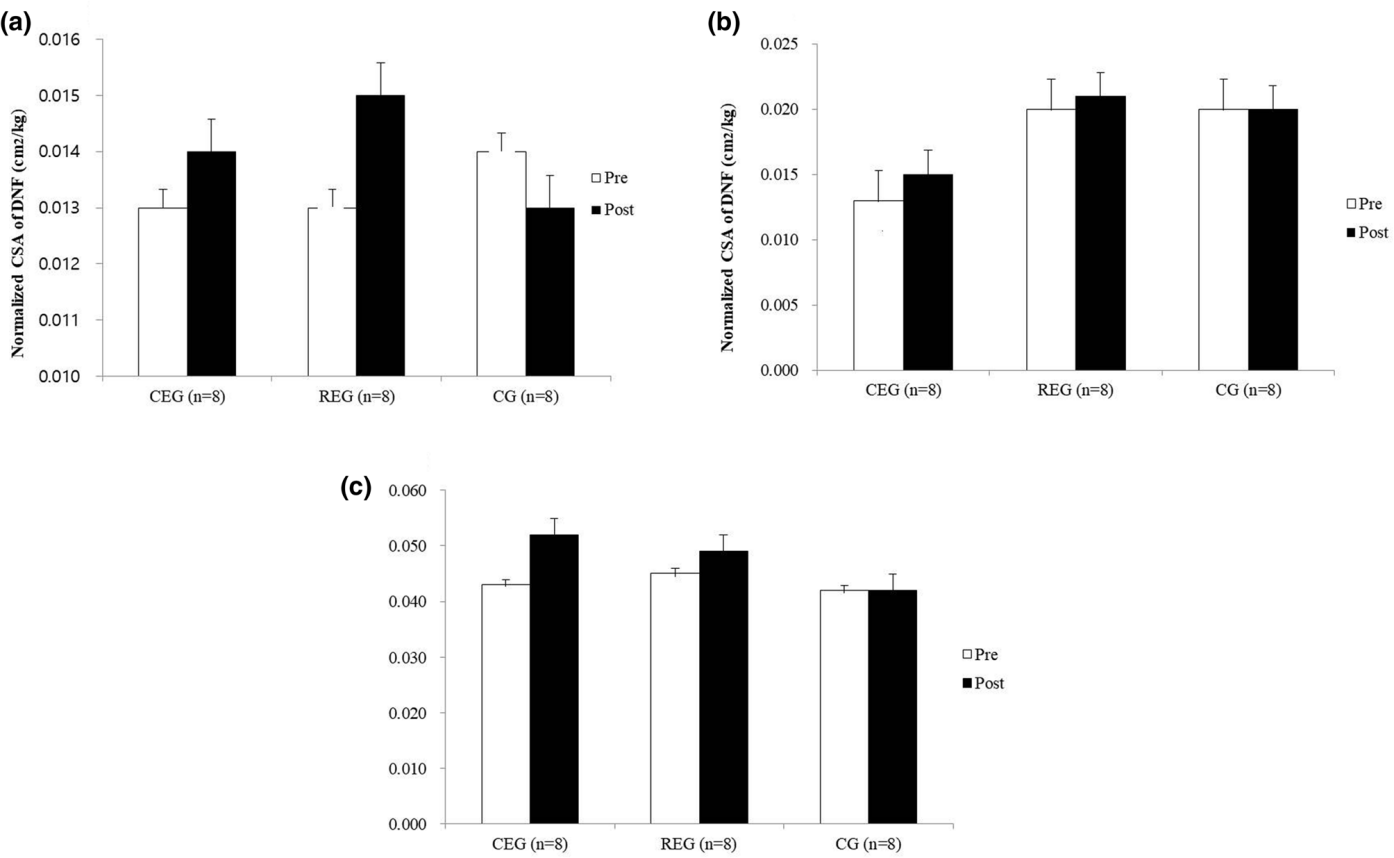
The difference of cross-sectional area in cervical deep muscles after intervention. (**a**) Longus colli muscle; (**b**) Longus capitis muscle; (**c**) Multifidus muscle. CEG, corrective exercise group; REG, resistance exercise group; CG, control group; CSA, cross-sectional area; DNF, deep neck flexor. *P* < 0.0167*, *P* < 0.00167**: the results from Bonferroni correction.

In change of CSA, Longus colli was not showed a significant different between three groups (*P* < 0.424). The change after intervention revealed in CEG (Wilcoxon signed-rank test, *P* < 0.012) but not showed in REG and CG (*P* < 0.134 and *P* < 0.401, respectively). There was a significant difference between three groups in change of longus capitis (Kruskal–Wallis test, *P* < 0.036) but not revealed a significant difference between CEG and CG (Mann–Whitney U test*, P* < 0.046), and REG and CG (*P* < 0.916), except CEG and REG (*P* < 0.016). In analysis of Wilcoxon signed-rank test, CEG and REG was showed a significant improvement after intervention (*P* < 0.012 and *P* < 0.012, respectively) but not showed a significant improvement in CG (*P* < 0.779). In change of multifidus CSA, there was a significant difference between the groups (Kruskal–Wallis test, *P* < 0.007). In post hoc test, the two active intervention, CEG and REG, has showed a significant different compared with CG (Mann–Whitney U test ; CEG vs. CG, *P* < 0.016 and REG vs. CG, *P* < 0.009, respectively) but not showed between CEG and REG (*P* < 0.074). The change after intervention revealed in CEG and REG (Wilcoxon signed-rank test, *P* < 0.012 and *P* < 0.012) but not showed in CG (*P* < 0.123) (Fig. 2). Table 2 shows the results after exercise intervention between the groups.

**Table 2:** The clinical outcomes after exercise intervention of 3 months between the groups.

Variable	Time	Two active interventions		One passive	*P*
		CEG	REG	CG	
Thoracic Cobb's angle (°)	Pre	42.00 ± 2.00	40.75 ± 1.83	39.63 ± 1.30	.000***
	Post	31.50 ± 2.78^†^	35.50 ± 2.29^†^	39.25 ± 1.04	
Maximal muscle strength(mmHg)	Pre	55.3 ± 88.02	65.13 ± 11.61	59.88 ± 21.04	.038*
	Post	82.13 ± 12.42^†^	76.63 ± 10.59^†^	58.38 ± 23.10	
Muscle endurance of 80% (sec)	Pre	10.63 ± 2.50	8.25 ± 2.82	10.13 ± 3.09	.000***
	Post	19.00 ± 2.73^†^	13.75 ± 2.82^†^	9.88 ± 2.17	
Muscle endurance of 50% (sec)	Pre	56.00 ± 8.04	50.63 ± 10.06	57.50 ± 6.23	.000***
	Post	80.88 ± 9.42^†^	63.88 ± 5.84^†^	56.75 ± 7.48	
Longus colli (cm^2^)	Pre	0.74 ± 0.12	0.83 ± 0.15	0.77 ± 0.16	.424
	Post	0.81 ± 0.13^†^	0.86 ± 0.15	0.76 ± 0.15	
Longus capitis (cm^2^)	Pre	0.75 ± 0.23	1.15 ± 0.19	1.14 ± 0.32	.036*
	Post	0.83 ± 0.23^†^	1.18 ± 0.21^†^	1.14 ± 0.32	
Multifidus (cm^2^)	Pre	2.48 ± 0.34	2.57 ± 0.23	2.37 ± 0.31	.007**
	Post	2.97 ± 0.46^†^	2.76 ± 0.19^†^	2.36 ± 0.30	

Mean ± standard deviation. CEG, corrective exercise group; REG, resistance exercise group; CG, control group.

^†^*P* < 0.05, ^††^*P* < 0.01, ^†††^*P* < 0.001: the results from Wilcoxon singed rank test.

*P < 0.05, **P < 0.01, ***P < 0.001: the results from Kruskal–Wallis test.

## Discussion

This study compared the effects of different exercise types on Cobb’s angle of the thoracic curve, as well as on the strength and endurance of cervical muscles, and the CSA of the cervical deep muscles in patients with thoracic hyperkyphosis. The corrective exercise program (CEP) was more effective in improving Cobb’s angle, cervical muscle strength and endurance, and CSA than resistance exercise program (REP) or CG..

Excessive thoracic hyperkyphosis is associated with misalignment of the spinal curve, resulting in forward head posture and cervical pain^1,4^. This study revealed that the corrective exercise and resistance training were beneficial interventions to improve thoracic hyperkyphosis angle. This result is supported by a study by Katzman et al.^6^ which reported that multidimensional group exercise for 12 weeks improved muscle strength and decreased thoracic Cobb’s angle by 6°. Additionally, previous systematic reviews^5^ also demonstrated that muscle strength exercise could improve thoracic Cobb’s angle in hyperkyphosis of the thoracic vertebrae. Particularly, there was a significant improvement in the thoracic kyphosis angle in CEG when compared to REG. The corrective exercise applied in this study was an integrated exercise program and included mobilization exercise and Schroth method, while resistance exercise only included weight training. Mobilization exercise and the Schroth method can reduce the angle of thoracic hyperkyphosis^7,13–15^. Foad et al.^11^ reported that corrective exercise is a more effective intervention than a general exercise program for patients with thoracic hyperkyphosis which supports the present findings. Therefore, corrective exercise, including mobilization and the Schroth method, is considered as an exercise intervention to improve thoracic Cobb’s angle in hyperkyphosis. These results explain why an integrated CEP is more effective than REP included only resistance training. However, further studies including patients with osteoporosis and vertebral fractures have to confirm if these results are applicable for the patients with osteoporosis, vertebral fractures, and kyphosis.

The thoracic spinal column functions as a supporting base for the cervical spine and influences cervical kinematics through the cervicothoracic junction^1^. Concomitant thoracic spinal motion is necessary to produce the complete range of movements at the cervical spine, which are often reduced with changes in the normal alignment of the thoracic spine^16^. An excessive thoracic kyphotic curve is associated with shortness of the cervical extensors and weakness of the cervical flexors^4^. In this study, CEG and REG showed improvement of cervical muscle strength and endurance after exercise therapy for 3 months, and these results are consistent with a study conducted by Kjellman et al.^17^ which reported that resistance training in patients with neck pain could improve static muscle strength and muscle endurance of the cervical muscles. Hakkinen et al.^18^ also reported similar results, showing an increase in cervical strength and endurance by applying resistance training. Both interventions included the same types of exercise, including back muscle strengthening exercises; however, CEP is an integrated program that includes stretching and mobilization. Several studies demonstrated that CEP is a beneficial intervention to improve Cobb’s angle and posture in thoracic hyperkyphosis^10,11,19,20^. According to those studies, the integrated exercise program could be considered as a specific exercise intervention to obtain more effective results with cervical muscle strength and endurance in patients with thoracic hyperkyphosis.

The cervical deep muscles, including the longus colli, longus capitis, and multifidus, have an important role in the control and stability of the cervical vertebrae^21^. Thoracic hyperkyphosis is associated with misalignment of the cervical curve and this could cause dysfunction of the cervical deep muscles, resulting in chronic cervical pain^4^. Patients with chronic neck pain were shown to have more severe deep muscle atrophy than healthy subjects^6^. Therefore, this study measured the CSA of deep muscles of the cervical vertebrae to compare the effects of different exercise types on CSA. According to the results of this study, the CSA of cervical deep muscles showed a significant improvement in the two active intervention groups. These results are similar to those of a study conducted by Cagnie et al.^22^ which demonstrated that cervical flexion exercises can increase the CSA of cervical deep muscles such as the longus colli and longus capitis. An improvement in CSA may be associated with improvement in muscle strength and endurance of the cervical deep muscles by applying cervical flexor strength exercises. Longus colli was only showed a significant improvement after intervention in CEG and it can be explained the CEP is superior intervention than REG and CG. The CSA of Longus capitis and multifidus were increased in CEG and REG but CEG was showed more improvement than REG. The explanation for this is that the decreased thoracic kyphosis contributes to reduce the overload at the cervical vertebrae and to increase cervical muscle activity, resulting in improvement of CSA in the cervical deep muscles. This mechanism can be explained on the basis of previous studies reporting the positive effects of Schroth and mobilization exercise on muscle activity^14,15^. We suggest that combined corrective exercise should be considered as a more efficient exercise type for increasing CSA of cervical muscles, compared with traditional resistance exercise.

This study has some limitations. Firstly, the subjects were all female. This aspect should be considered because the prevalence of hyperkyphosis in women or men could influence the results. Therefore, this result is not generalizable to all populations, and further study is needed to confirm the difference between male and female subjects. Secondly, the study had a small sample size although it was divided into three different groups. Thus, it is difficult to apply these results to all patients with thoracic hyperkyphosis. A large sample study is needed to confirm these results. Third, this study was not measured the strength of back muscles despite of back strengthening exercise was applied in two exercise groups.

In conclusion, corrective and resistance exercises are beneficial interventions for improving thoracic Cobb’s angle, cervical muscle strength and muscle endurance, and the CSA of the cervical deep muscles. These exercises yield better results compared with simple physical therapy. This study revealed that a CEP is a more effective intervention than traditional resistance exercise for improving these parameters. Therefore, these exercise types should be considered when exercise intervention is prescribed to patients with thoracic hyperkyphosis.

## Methods

### Study design

This study was a randomized controlled trial with single blind in the participants. It included 38 female patients with thoracic hyperkyphosis with chronic cervical pain diagnosed by an orthopedist. The diagnostic criteria were as follows: chronic neck pain on cervical posterior and upper trapezius muscles for 6 months, kyphosis angle ≥ 38° of Cobb’s angle, and a score > 7 on the visual analog scale concerning cervical pain. Of these patients, 14 were excluded due to no exercise recommendation from the doctor since three subjects had disc herniation and three were diagnosed with osteoporosis and eight patient refused participation in this study. Twenty-four patients were recruited and informed consent was obtained. Inclusion criteria were: participants having no surgical history within the last three months, no orthopedic and neurological problems, and no experience with exercise therapy for thoracic kyphosis or neck pain. The participants were randomly allocated according to their order of registration in the study to three groups, namely two active intervention groups [the CEG (n = 8) and REG (n = 8)], and one passive group [CG (n = 8)]. The study protocol was approved by the Sunmoon University Institutional Review Board (IRB No. SM-201603–004-2) and complied with the 1964 Helsinki declaration and its later amendments.

### Outcome assessments

A baseline assessment was conducted before randomization and re-assessment was performed after 12 weeks using the same method. The primary outcomes were Cobb’s angle, muscle strength, and endurance of the cervical muscle. The secondary outcome was the CSA of the cervical deep muscles including the longus colli, longus capitis, and multifidus muscles.

### Cobb’s angle

**Figure 3:**
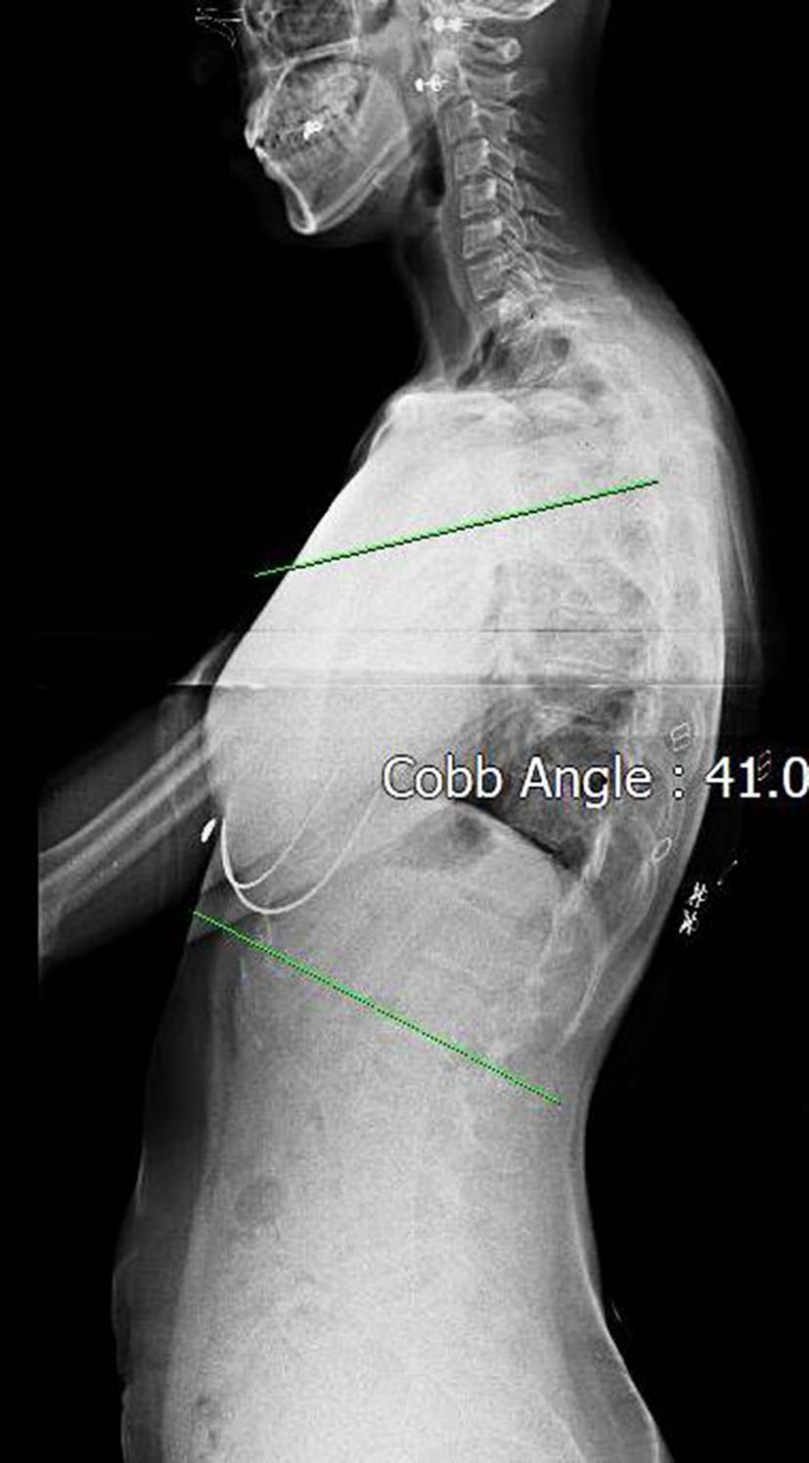
The measurement of Cobb’s angle using picture achieving and communication system (PACS).

The change in Cobb’s angle was measured by a professional radiologist at a local medical hospital. Participants were instructed to stand with a straight chest and back, with both arms crossed on the chest, and X-rays were taken in the sagittal view. Cobb’s angle was measured using Cobb’s method, which involves drawing a parallel line at the upper thoracic endplate of the second thoracic vertebrae and lower thoracic endplate of the twelfth thoracic vertebrae, and then erecting perpendiculars from these lines to cross each other^23^ (Fig. 3).

### Muscle strength and endurance

Changes in muscle strength and endurance were assessed using a Stabilizer Pressure Biofeedback unit (Stabilizer pressure, Chattanooga, USA). The subjects lay comfortably with knee flexion of 90°, and the device was placed and on the upper cervical spine. Participants were instructed their jaw to push down onto the neck and to hold the resistance in the craniocervical flexion direction against the handheld dynamometer. The reference pressure was set at 40 mmHg, which was the average pressure at which the subject could contract the deep neck flexors without discomfort. The maximum cervical strength and endurance were measured as the time to maintain 80% and 50% of maximal muscle strength^24^. Trials of the maximum cervical muscle strength were conducted 3 times with 30-s rest time between each trial. After resting for 1 min, the subject was instructed to maintain 80% of the maximum strength within 84.24 mmHg for CEG, 92.10 mmHg for REG, and 87.90 mmHg for CG. This was measured three times. The subject tried the same method at 50% of maximum strength within 67.65 mmHg for CEG, 72.57 mmHg for REG, and 69.94 mmHg for CG after resting for 1 min. The subject did not complain of pain during the test. The measurement was stopped when the error of the pressure gauge was within ± 2 mmHg, and recorded that time. The average value of the three measurements was used for the data analysis.

### Cross-sectional area of the cervical deep muscles

**Figure 4:**
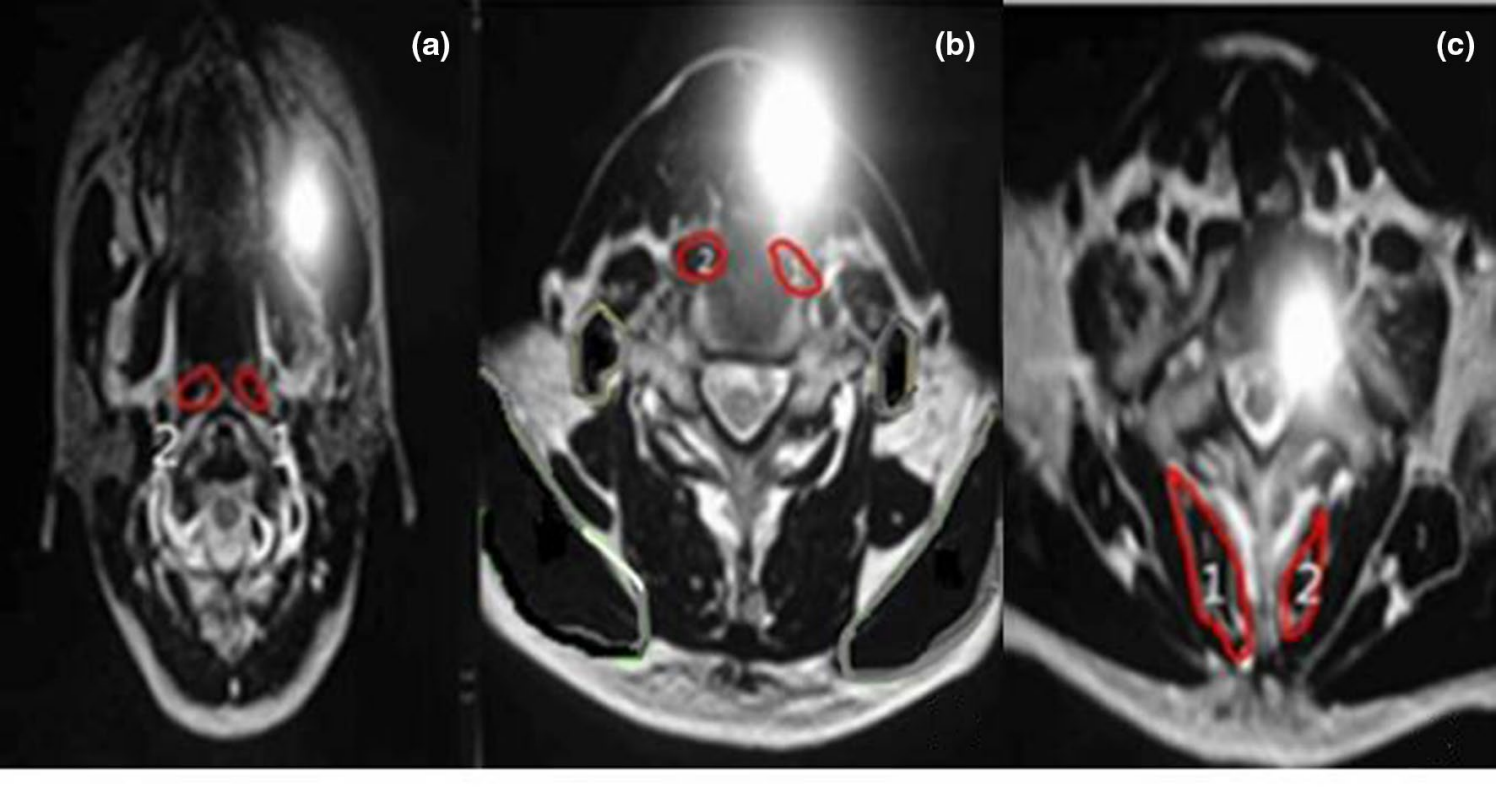
MRI (magnetic resonance imaging) imaging of the cross-sectional area of the perivertebral space. (**a**) Longus colli muscle; (**b**) Longus capitis muscle; (**c**) Multifidus muscle.

The CSA of the cervical deep muscles was measured using 1.5 T magnetic resonance imaging (Intra, Philips, Netherlands) before and after intervention. To measure the CSA of the cervical muscles, a T2 image was obtained and used by cutting the transverse axis between the upper and lower endplate of the 5th to 6th cervical vertebrae at 5 mm^2^ thickness and 10 mm^2^ interval. The image was analyzed using a picture archiving and communication system by an experienced radiologist of 10 years to enhance intra-reliability of the measurement. The CSA of the muscles were calculated by setting and drawing the regions of interest to the right and left of the cervical vertebrae muscles. The right side was used for the analysis (Fig. 4).

### Interventions

In this study, correction and resistance exercises were applied to the two active intervention groups, and the passive group was CG. The total exercise time was 60 min including 5 min of warm-up, 50 min of the main exercise, and 5 min of cool down. Each session was conducted 3 times a week for 12 weeks. The CEP was a modified program from previous studies^11,14^. The CEP is an integrated intervention that includes stretching, mobilization, and Schroth method, which is different with REP. However, the CEP and REP consisted of the same back extensor training. This program focused on a decrease in the thoracic kyphosis angle, improvement of lung function and enhancement of respiratory muscle strength through chest or abdominal breathing training, and improvement of neck pain and function. The exercise intensity was set using a rating perceived exertion scale, which ranged from 13 (somewhat hard) to 15 (hard). The resting time was 30 s between each set and 60 s between exercise items. The REP was applied by modifying several resistance programs reported^13,16^. The exercise intensity was measured by evaluating one repetition maximum (1 RM) for each exercise, and the intensity was set at 30% of 1 RM. The resting time was applied in the same way as that of the CEP. The intervention for CEG was applied CEP while REG was conducted REP (Table 3).

**Table 3:** The exercise program was divided into two active intervention groups including corrective and resistance exercise.

Exercise types	Exercise modes	Time	Intensity
**Corrective exercise program**			
Cervical corrective exercise	Cervical retraction exercise (4 min) Cervical mobilization exercise (4 min) Cervical flexion exercise (4 min)	12 min	20R / 3Sets RPE 13–15
Thoracic corrective exercise	Thoracic extension exercise (4 min) Thoracic mobilization exercise (4 min) Scapula adduction exercise (3 min) Shoulder horizontal abduction exercise (3 min)	14 min	20R / 3Sets RPE 13–15
Back extensor exercise	Trunk extension exercise (3 min) Arm and leg raise exercise (3 min)	6 min	20R / 3Sets RPE 13–15
Latissimus dorsi and pectoralis stretching	Foam roller stretching (3 min) Gymball stretching (3 min)	6 min	20R / 3Sets RPE 13–15
Schroth method exercise	Kneeling between chair (4 min) Standing in a doorway (4 min) Sitting question mark (4 min)	12 min	20R / 3Sets RPE 13–15
Warm-up and cool down	Stretching for lower and upper body	10 min	RPE 13
**Resistance exercise program**			
Cervical resistance exercise	Cervical retraction with thera-band (4 min) Cervical bridge exercise (3 min) Cervical lift off exercise (3 min)	15 min	1RM of 30%
Shoulder and scapular muscles strengthen exercise with thera-band	Scapular retraction (4 min) Lat pull-down (4 min) Seated low rowing (3 min) Prone lying with arm elevator (4 min)	15 min	1RM of 30%
Posture exercise	Back extension with weight backpack (5 min) Four point kneeling (5 min)	10 min	1RM of 30%
Leg strength exercise	Half squat (5 min) Step ups (5 min)	10 min	1RM of 30%
Warm-up and cool down	Stretching for lower and upper body	10 min	RPE 13

*RM* repetition maximum, *RPE* rating of perceived exertion.

The CG with one passive group was treated by the therapy consisted of thermal stimulation, using an electric heat pack, and ultrasound therapy. The thermal therapy was applied using an electric heat pack for 25 min by setting the temperature at 50° to 55°, and ultrasound therapy used an Inter current therapy (ITO, Japan) for 15 min at 100 Hz, and a frequency of 1 MHz and intensity of 1.5 W/cm^2^ for 10 min, with a total treatment period of 50 min.

### Statistical methods

The Kolomgrov-Smirnov test was used to check normal distribution of the data. The baseline characteristics of the three groups were compared using Kruskal–Wallis test, and the mean and standard deviation were used for the descriptive statistics of all the variables in the groups. Kruskal–Wallis test was used to confirm the different effects of each exercise type. The Mann–Whitney U test was used when a significant difference was confirmed and Bonferroni correction was applied with the alpha level set at *P* = 0.0167. The Wilcoxon signed-rank test was used to compare all the variables between the pre-intervention and the post-intervention. All the data were analyzed using Statistical Package for Social Sciences (SPSS) 18.0 statistics program for Windows. *P* values < 0.05 were considered statistically significant.

## Data Availability

The datasets generated during and/or analyzed during the current study are not publicly available due to the institutional ethical regulations of Sunmoon University but are available from the corresponding author on reasonable request.
